# Functional genomics of the lactic acid bacterium *Limosilactobacillus fermentum* LAB-1: metabolic, probiotic and biotechnological perspectives

**DOI:** 10.1016/j.heliyon.2022.e11412

**Published:** 2022-11-05

**Authors:** Tanim Jabid Hossain

**Affiliations:** a Department of Biochemistry and Molecular Biology, University of Chittagong, Chattogram, Bangladesh; b Biochemistry and Pathogenesis of Microbes (BPM) Research Group, Chattogram, Bangladesh

**Keywords:** *Limosilactobacillus fermentum*, Probiotic lactic acid bacteria, Functional genomics, Metabolomics, Nutraceuticals, Exopolysaccharide, Antimicrobial peptide, Functional food

## Abstract

A genome-based systematic analysis was conducted to characterize the metabolic, probiotic, fitness, and safety properties of *Limosilactobacillus fermentum* LAB-1, a lactic acid bacterium demonstrating strong antimicrobial effects against clinical pathogens. Gene functional characterization revealed a large number of genes for carbohydrate metabolism and a heterofermentative system for carbon dissimilation. Genes for intact pyruvate oxidation, pentose phosphate, and PRPP biosynthetic pathways were identified. Substantial carbohydrate-active enzymes and transporters were also predicted. Metabolic reconstruction revealed complete sets of enzymes for arginine, lysine, methionine, threonine, proline, and ornithine biosynthesis. The bacterium harbors a diverse range of peptidases, and a large variety of peptide and amino acid uptake systems. It encodes restriction-modification and CRISPR-Cas systems for protection against phage infections and carries a wide spectrum of stress proteins for adaptation in the gut and industrial conditions. Genes related to the biosynthesis of B-group and K vitamins were identified allowing its application for novel bio-enriched food production. Other beneficial traits of probiotic and industrial importance such as production of flavor compounds, exopolysaccharide, acetoin, and butanediol were identified. Three antimicrobial peptides were predicted which showed >98% sequence-identity to experimentally validated bacteriocins. Negative traits such as transmissible antibiotic resistance, pathogenicity or virulence appeared to be absent suggesting the strain to be considered safe. The genome analysis will allow precisely targeted laboratory research and full exploitation of the probiotic potentials towards functional-food, biotechnology and health-related applications.

## 1 Introduction

Lactic acid bacteria (LAB) are a large diverse group but have in common that they are Gram positive, catalase negative, non-spore-forming, and strictly fermentative microbes which produce lactic acid as the major end-product of sugar fermentation [1, 2]. Species belonging to this group play important functions in food fermentation and animal nutrition with some species being highly beneficial to human health [3]. These bacteria are usually fastidious and show a complex nutritional requirement [4] because they lack several biosynthetic genes such as those required for some essential amino acids, nucleotides and vitamins [5]. Accordingly, LAB are mostly found in nutrient-rich environments wherefrom they can readily obtain the essential nutritive elements. LAB were often recovered from vegetables, fruits, flowers, and mucosal surfaces of human and animals. They were also isolated from a variety of food products especially fermented meats, vegetables, prickles, beverages, dairy products, silage, and sour dough [6, 7]. Some LAB are also used in food preparation to initiate desired changes [8]. The autochthonous LAB naturally present in foods or the non-native LAB deliberately added during food processing may both provide health promoting effects. Most health benefits associated with LAB are strain specific [9, 10, 11] which include improved digestion and nutrition, prevention and control of infections, control of certain cancers, regulation of serum cholesterol etc. [12, 13]. Besides, certain LAB strains demonstrate an enhanced ability to produce nutraceuticals, compounds that provide physiological benefits, for example, vitamins, exopolysaccharides (EPS), bacteriocins, natural products etc. [14, 15]. Production and release of these beneficial microbial compounds can largely increase foods’ nutritional values.

*Limosilactobacillus fermentum* is an important lactic acid bacterium with some probiotic activities. It is recognized as a safe bacterium and used in food preparation to trigger fermentation [16, 17]. It is found in human milk, gastrointestinal tract and oral cavity, and also isolated from natural foods and food products. The bacterium and its probiotic properties have been a subject of rigorous research in the recent years [16]. Strains of this LAB offer technological benefits such as an enhanced flavor, texture, and aroma of food products, as well as probiotic benefits such as anti-infectious, anti-inflammatory, immunomodulatory, pro**-**longevity, and damage preventive effects [16, 18]. The bacterium also exhibited good adherence ability to intestinal mucosa [19]. However, variations in the above properties were observed across strains.

*L. fermentum* LAB-1 was isolated recently from the popular dairy beverage borhani [20, 21]. The strain has potent antimicrobial activity exhibited against several clinically relevant bacterial pathogens including both Gram-positive and Gram-negative taxa such as *Acinetobacter baumannii*, *Bacillus cereus*, *B. subtilis*, *Escherichia coli*, *Klebsiella pneumoniae*, *Pseudomonas aeruginosa*, Salmonella abony, Salmonella typhi, *Shigella flexneri*, and *Staphylococcus aureus* [20]. Currently, the strain is under further in-vitro and in-vivo studies towards elucidation of its additional probiotic effects. Its genome sequence of 2,011,628 nucleotides having 2,081 genes and 1,913 proteins has been deposited (Bioproject: PRJNA786104). In this work, functional analysis of the genome is undertaken to characterize its metabolic features and to identify its strain**-**specific genes of probiotic and biotechnological relevance, stress-responsive genes required for adaptation in gut and dairy niches, and defense-system genes providing immunity against invading DNA. Strain safety was assessed based on the presence of genes encoding antimicrobial resistance (AMR), pathogenic and virulence factors (VF). The genome analysis provides the genetic foundation towards full understanding of the strain's metabolic, probiotic and safety aspects for functional food, biotechnology, therapeutic exploitations.

## 2 Materials and methods

### 2.1 Bacterial strain

The LAB**-**1 strain was isolated from borhani and taxonomically classified based on the analysis of average nucleotide identity (ANI) of its genome sequence by the Prokaryotic Genome Annotation Pipeline (PGAP) of NCBI [22, 23]. PGAP annotation revealed that LAB-1 strain has 99.37 ANI to *L. fermentum* ATCC 14931, therefore classified as *L. fermentum*.

### 2.2 Genome data

The genome data was submitted to GenBank under the accession number JAJTII000000000.1.

### 2.3 Functional annotations and categorization

Cellular functions of the proteins encoded in the genome and their functional categorization were inferred using BlastKOALA against the Kyoto Encyclopedia of Genes and Genomes (KEGG) database. The proteins’ amino acid sequences were extracted from the annotated coding sequences and searched against KEGG Orthology (KO) ID and Clusters of Orthologous Groups (COG). The Rapid Annotations using Subsystems Technology (RAST) was also used for the functional annotation as well as for the function-based, sequence-based or KEGG-based comparison of metabolic reconstructions [24, 25]. Additionally, if deemed necessary to further validate the functional annotations, some of the predicted gene sequences were also examined by NCBI BLAST searches confirming their identity [26].

### 2.4 Comparison of orthologous gene clusters

The analysis and comparison of whole genome orthologous gene clusters of LAB-1 with other strains was carried out using their protein sequences in OrthoVenn2 [27].

### 2.5 Determination of metabolic and probiotic features

Metabolic features and pathways were determined based on the Kegg Orthology (KO) system [28]. The KO number that was assigned by BlastKOALA to each of the protein sequences in the genome was submitted to Kegg Mapper to reconstruct the metabolic pathway [29]. The KO numbers were also used to generate metabolic pathways using the iPath3.0 module [30].

### 2.6 Identification of carbohydrate active enzymes

Predicted gene sequences were subjected to the identification of carbohydrate active enzymes as described previously using the following parameters: HMMER (E-value < 1e−15, coverage >0.35), DIAMOND (E-value < 1e−102), eCAMI (important_k_mer_number ≥ 5, k_mer ≥ 8) [31]. Information about CAZyme families was collected from the CAZy database.

### 2.7 Identification anti-phase defense systems

The restriction-modification (R-M) system, and the CRISPR-Cas system with associated direct repeats (DRs) and spacers were identified based on [32] and functional annotations. Identification of prophage genes was implemented using the phage search tool enhanced release [33].

### 2.8 Identification of antimicrobial peptides

Antimicrobial peptides were identified based on sequence similarity using protein BLAST of query sequences against a custom database of the LAB-1 proteins executed in NCBI BLAST suite standalone version 2.13.0 or by RAST. Accession numbers of the query sequences are BAG27613.1, ARB00861.1, and EEI22241.1.

### 2.9 Analysis of safety aspects

Presence of antimicrobial resistance was analyzed using ResFinder version 4.1 and the Comprehensive Antibiotic Resistance Database (CARD; McMaster University, Hamilton, Ontario, Canada) version RGI 5.2.1, CARD 3.2.0 [34,35]. Pathogenicity and virulence of the isolate in human hosts were analyzed by PathogenFinder 1.1 and VirulenceFinder 2.0 respectively using the isolate's proteome or contig sequences according to [36, 37].

## 3 Results and discussions

### 3.1 Overview of functional annotations

**Figure 1:**
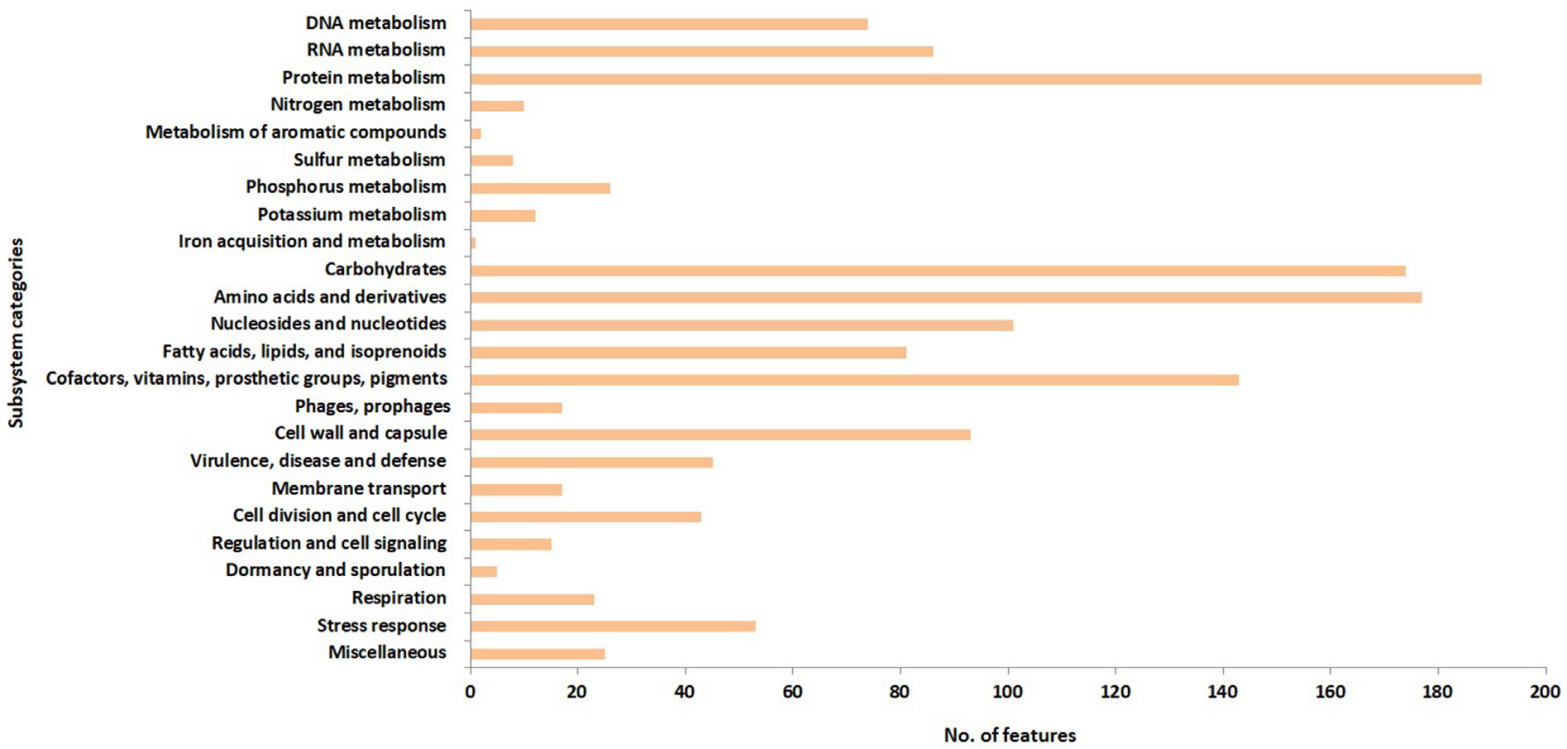
Functional annotation of the predicted coding sequences of LAB-1 strain to various cellular and metabolic processes based on KEGG database.

Functional genome analysis identifying genes in an organism with functional characterization is essential to understanding of the organism's cellular and metabolic processes, and elucidation of its beneficial features for probiotic and industrial applications. The functional annotation of LAB-1 genome was implemented by Rapid Annotations using Subsystems Technology (RAST) and BlastKoala. RAST identifies and assigns coding sequences (CDSs) to a collection of functionally related protein-families called the subsystems that are housed within the SEED database. A total of 1,914 CDSs were predicted by RAST that were distributed across 313 SEED subsystems and 1157 cellular features. A major proportion of the cellular features was assigned to protein metabolism (188) followed by amino acids and derivatives (177), carbohydrates (174), cofactors, vitamins, prosthetic groups and pigments (143), nucleosides and nucleotides (101), and other functional categories (Figure 1). The genes associated with protein metabolism were dominated by those of protein biosynthesis (158), whereas genes linked to carbohydrates were dominated by those of the central carbohydrate metabolism (59), di- and oligosaccharides (26), organic acids (23), and fermentation (20). A full list of the subsystems and functional features identified in LAB-1 is provided in the Supplementary ∗∗S1.

**Figure 2:**
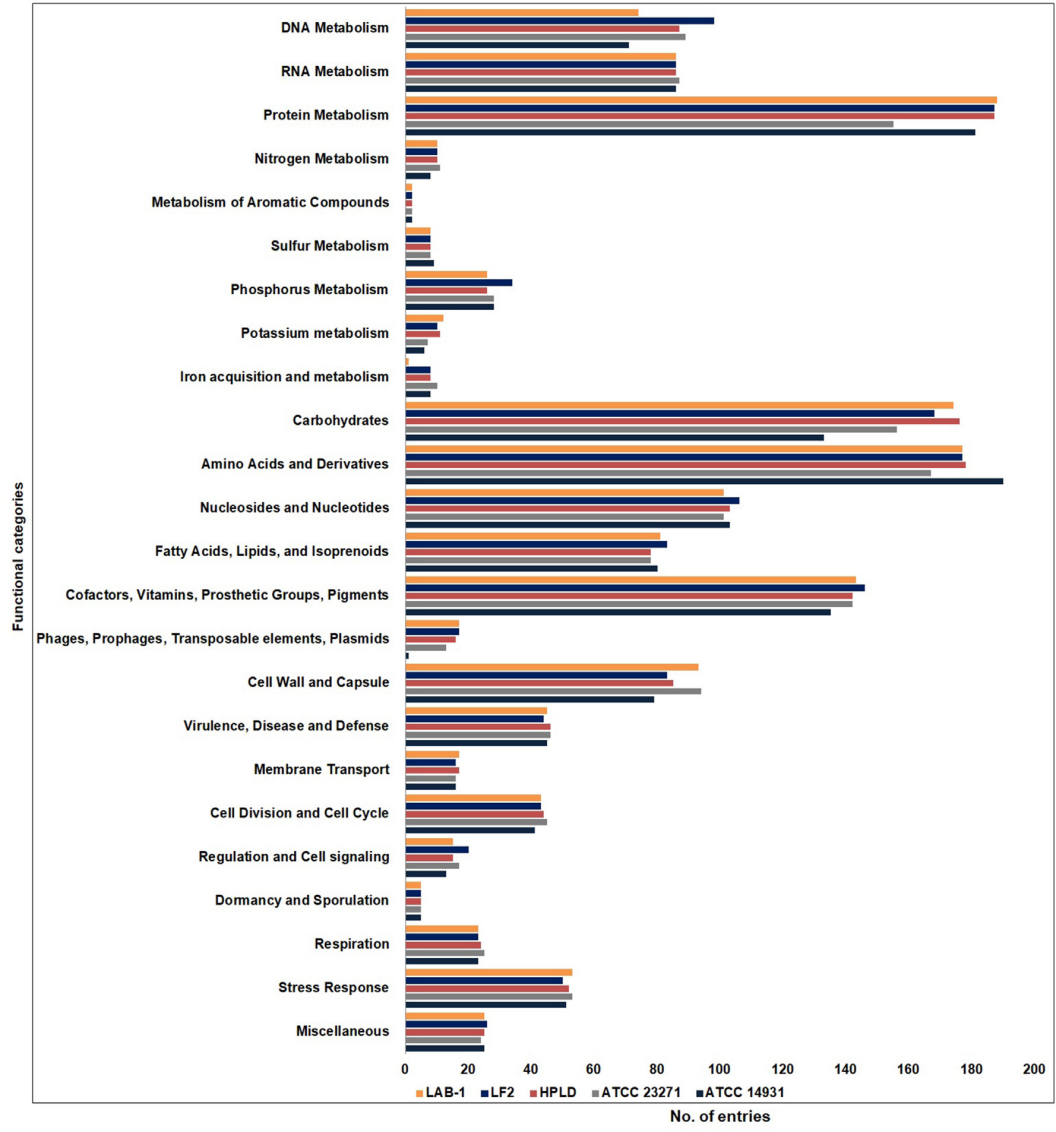
Distribution and functional categories of the RAST-predicted protein-coding genes identified in the genomes of five *L. fermentum* strains, LAB-1, ATCC 23271, ATCC 14931, HPLD and LF2.

Although RAST offers a consistent and accurate genome annotation, the design and implementation of the annotation are conceptually different from that of BlastKoala [24]. Therefore, the functional annotation was also executed by the later using predicted protein sequences of the genome. BlastKoala assigned 1161 from a total of 1913 entries to various cellular processes and categorized them into 23 functional groups (Figure 2; orange bar). According to this annotation, the most prevalent metabolic pathway was that of the carbohydrates and the most prevalent protein family was the one involved in genetic information processing. Reconstruction of the entries by KEGG Mapper sorted them into 172 different biological processes, 35 groups of genes and proteins, and 23 metabolic pathway modules, presented in the Supplementary ∗∗S2.

Consistent with these results, the functional genome analysis of other *L. fermentum* strains, for example, ATCC 23271 and ING8 also showed similar annotation properties with their 2123 and 2103 coding sequences distributed across 312 and 208 subsystems respectively [38, 39]. Moreover, similar to LAB-1, a large proportion of the subsystem features was dedicated to protein metabolism, amino acids and derivatives, and carbohydrates.

### 3.2 Comparative genome and metabolome analysis

**Figure 3:**
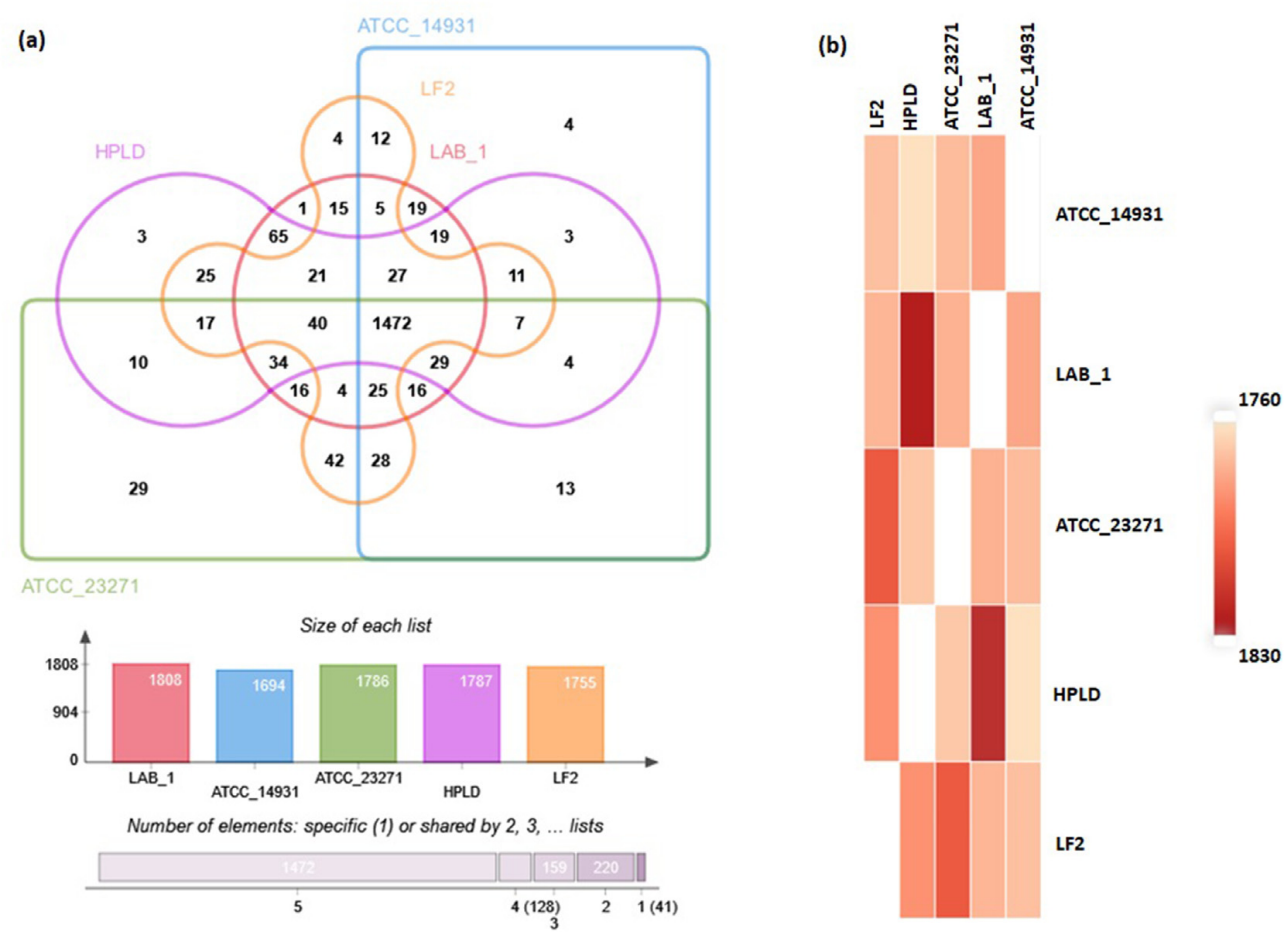
Orthologous gene clusters among various *L. fermentum* strains. (a) Venn diagram and bar charts comparing the number of shared and unique clusters of orthologous genes among *L. fermentum* LAB-1, ATCC 23271, ATCC 14931, HPLD and LF2. (b) Heatmap showing intra-strain similarities of orthologous subsets.

**Figure 4:**
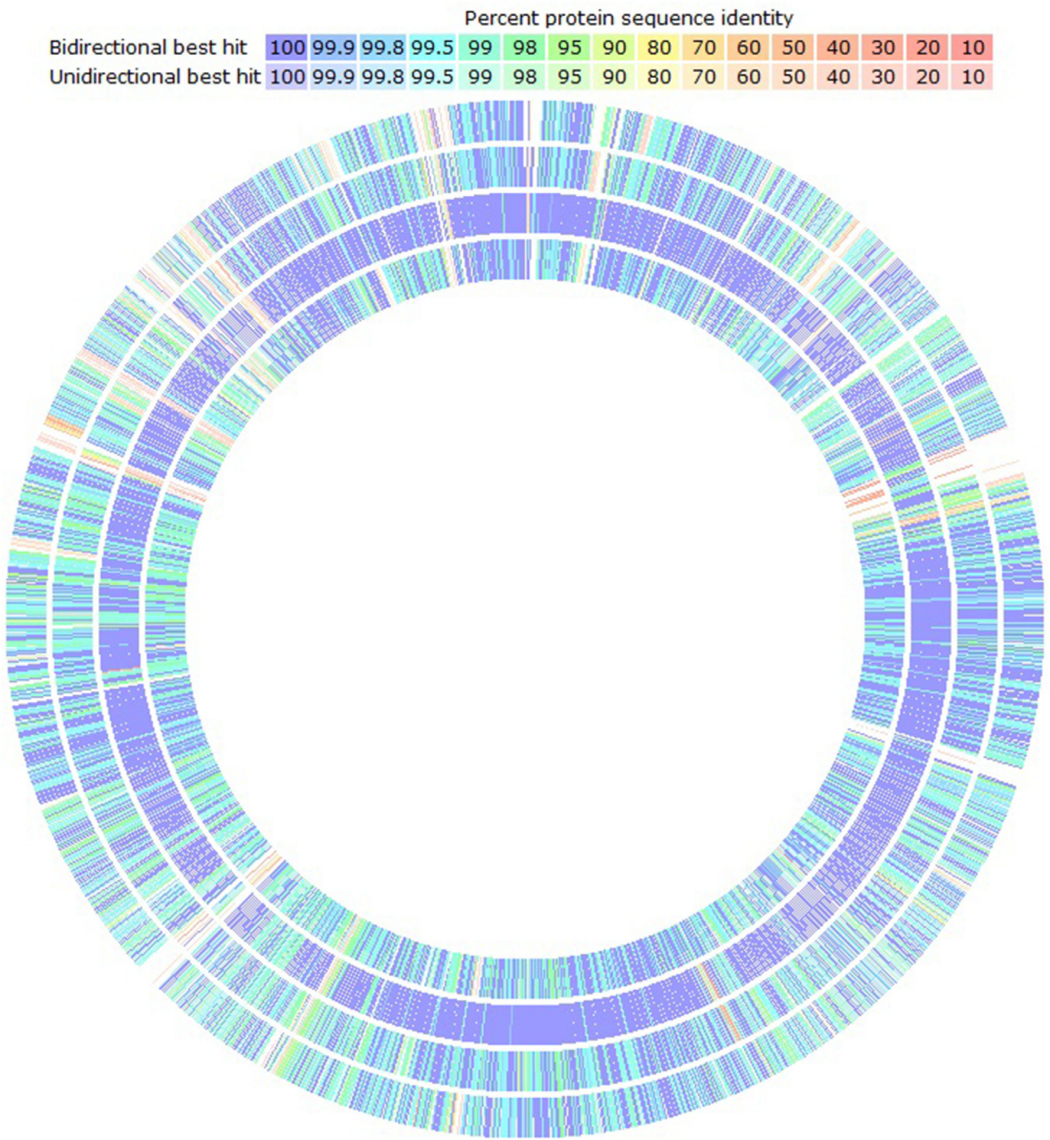
Sequence based genomic comparison of the *L. fermentum* strains. From outer to inner rings: ATCC 14931, ATCC 23271, HPLD, and LF2. Percent identity of protein sequences in reference to those in LAB-1 represented by color codes from purple (100% identity) to red (0% identity).

The LAB-1 genome was compared to that of four other *L. fermentum* strains with regards to their orthologous genes, and the metabolites encoded. The four strains are: (1) ATCC 23271 which has been reported to have inhibitory effects against Candida spp. [40]; (2) ATCC 14931, the genome of which is designated as the reference genome for Human Microbiome Project; (3) HPLD, a strain isolated from human feces; and (4) LF2 isolated from cheese. A genome-wide analysis of orthologous clusters was implemented using the predicted protein sequences which revealed that the five strains formed a total of 2020 protein clusters. The core of the genomes was found to be consisted of 1472 orthologous proteins shared by all the five strains indicating their conservation in the lineage after speciation (Figure 3a). The large set of proteins in the core genome that accounts for about 73% of the total clusters suggested a high similarity across the strains. In the LAB-1 strain, 1808 of the 1913 proteins formed clusters whereas 91 proteins remained as singletons. As the pairwise heatmap demonstrates, it shares the highest number of orthologous proteins (1824; 95.3%) with the HPLD strain, followed by ATCC 14931 (1778; 93%), ATCC 23271 (1775; 92.8%) and LF2 (1774; 92.8%) (Figure 3b). The functional categories were also compared among the genomes which showed similar distributions of metabolic features across the five strains (Figure 2). However, the LAB-1 genome was dominated by additional genes for carbohydrates, cell wall and capsule but encoded fewer genes for amino acids and derivatives. The higher number of genes for sugar metabolism may reflect its adaptation to the environment rich in carbohydrates which is consistent with its source of isolation. A further comparison based on protein sequence identity is presented in Figure 4 which also showed a high similarity among the strains. Some low-similarity regions were also detected. Most proteins in the low-similarity regions were phage proteins and hypothetical proteins, and a few glycosyltransferases and transcriptional regulators.

### 3.3 Sugar metabolism

**Figure 5:**
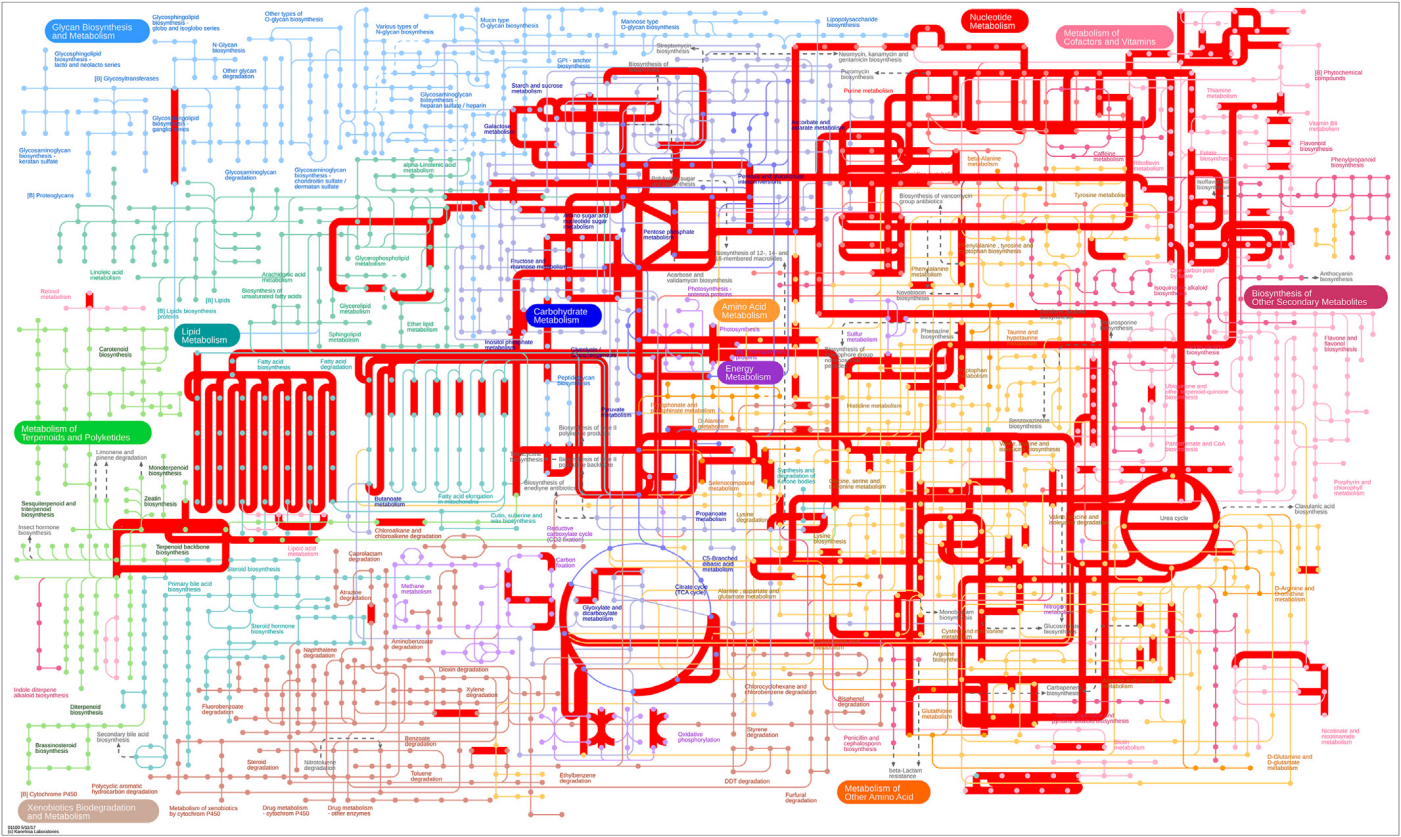
The Kyoto Encyclopedia of Genes and Genomes (Kegg) metabolic networks in LAB-1. The pathways were generated based on the KEGG orthology numbers. The metabolic pathways that are present in LAB-1 genome are indicated by thick red lines.

A comprehensive overview of the metabolic pathways generated using the KO numbers is presented in Figure 5. Pathways of the central carbohydrate metabolism including glycolysis, pyruvate oxidation, pentose phosphate pathway, PRPP biosynthesis, and gluconeogenesis were found to be present. Although the strain carries all genes required for reconstruction of the intact pyruvate oxidation pathway (4 genes), pentose phosphate pathway (12 genes), and PRPP biosynthetic pathway (2 genes), two genes appeared to be missing in the glycolytic (Embden-Meyerhof) pathway (Supplementary ∗∗S3). Enzymes of the glycolytic core module involving the three-carbon compounds were all identified (one copy of glyceraldehyde 3-phosphate dehydrogenase, EC:1.2.1.12; one copy of pyruvate kinase, EC:2.7.1.40; one copy of phosphoglycerate kinase, EC:2.7.2.3; one copy of enolase, EC:4.2.1.11; two copies of triosephosphate isomerase, EC:5.3.1.1; one copy of glucose-6-phosphate isomerase, EC:5.3.1.9; and two copies of 2,3-bisphosphoglycerate-dependent phosphoglycerate mutase EC:5.4.2.11). However, two key enzymes, fructose-bisphosphate aldolase and phosphofructokinase, were absent which suggested that the LAB-1 strain is an obligate heterofermenter and exclusively employs the phosphoketolase pathway (PKP), a unique route of glycolysis for carbon dissimilation [41, 42]. The PKP pathway converts glucose-6-phophate into acetyl-phosphate and glyerone-3-phosphate via phophoketolase, and produces large amounts of acetic acid and ethanol as byproducts in addition to lactic acid [43]. These substances possess antimicrobial activity which might be one of the reasons behind the strong inhibitory effect of LAB-1 strain reported against several clinical pathogens [20]. The above byproducts of the PKP pathway also impart taste and flavor to food products thus improving the sensory properties. Moreover, the lactic acid bacteria that are obligately heterofermentative are known to have several important health promoting effects such as immunomodulatory activities, antioxidant activities, improvement of the gut flora etc. [41].

The other carbohydrate metabolic pathways such as uptake and utilization of both lactose and galactose were identified including all genes for the intact Leloir pathway (galactokinase, EC:2.7.1.6; UDPglucose-hexose-1-phosphate uridylyltransferase, EC:2.7.7.12; UDP-glucose 4-epimerase, EC:5.1.3.2; aldose 1-epimerase, EC:5.1.3.3). The ability to metabolize lactose and the resulting galactose is an important criterion for starter selection [44] which makes the LAB-1 strain particularly suitable for use in starter culture.

Several transporters mediating carbohydrate uptake were also detected. Further details on the sugar transporters are provided under the “Transport systems” section below.

### 3.4 Carbohydrate active enzymes

To gain further insights into the utilization of carbohydrates, genes encoding Carbohydrate-Active enZyme (CAZymes) were identified. CAZymes are the proteins that create, modify, or degrade glycans and glycoconjugates [45]. They are stored in the database called “CAZy” that classifies all CAZymes across organisms. In the LAB-1 genome, 92 genes were detected that encode CAZymes, 40 of which belonged to the glycoside hydrolase (GH) family, 37 belonged to glycosyltransferases (GTs), 13 carbohydrate-binding modules (CBMs), and 2 auxiliary activities (AAs) (Supplementary ∗∗S4). The identified GHs were very diverse and distributed across sixteen families (GH1, GH2, GH5, GH13, GH23, GH24, GH25, GH28, GH32, GH36, GH38, GH42, GH65, GH73, GH84, and non-classified) but majority belonged to the GH13 family (27.5%), the largest in the CAZy database. Enzymes in the GH13 family mostly include hydrolases, transglycosidases, and isomerases with closely related activities and act on substrates containing α-glucoside linkage such as starch, glycogen, and related oligo- and polysaccharides [46]. The GTs identified in the genome were distributed across ten families (GT2, GT4, GT28, GT30, GT51, GT58, GT83, GT111, GT113 and non-classified) but mostly dominated by enzymes from GT2 and GT4 (37.8% each). These two families contain a large number of GTs which utilize diverse nucleotide-sugar donors in the glycosidic bond formation [47, 48]. Among the identified CBMs, majority belonged to the CBM50 group (84. 6%). These modules consist of about 50 residues and found connected with various GH enzymes.

### 3.5 Amino acid metabolism

The complete set of enzymes for the biosynthesis of arginine, lysine, methionine, threonine, proline, and ornithine was encoded in the LAB-1 genome. But one or a few enzymes in the biosynthetic pathways of some essential amino acids such as leucine, isoleucine, valine, histidine, tryptophan, and phenylalanine were missing. Hence, LAB-1 appeared to be auxotrophic for all or some of these amino acids. Biosynthesis of these amino acids might be carried out using intermediates produced in other pathways such as fatty acid metabolism or tricarboxylic acid cycle, or with low efficiency using the enzymes of fatty acid biosynthesis, or the bacterium can acquire the amino acids directly from the degradation of exogenous proteins [49]. Indeed, a battery of genes encoding a diverse range of peptidases was identified in the genome. These include peptidases from five major groups: aspartic, cysteine, serine, glutamic and metallo peptidases (Supplementary ∗∗S5). The peptidases together with a plenty of amino acid permeases and transporters (described in the following subsection) could help with the deficiency of the above amino acids through efficient processing and recovery from nutritionally rich environmental sources [50].

Previously in a comparative genome analysis of 28 *Lactobacillus fermentum* strains, biosynthetic pathways of aspartate from oxaloacetate; asparagine, lysine, and threonine from aspartate; glycine from serine; glutamine from glutamate; glutamate from glutamine; and cysteine from alanine and vice versa; were found to be present in the extended core genome, suggesting that these amino acids are non-essential for those strains [51]. In contrast, biosynthesis of methionine from aspartate and cysteine; proline from glutamate or ornithine, and arginine from glutamate and aspartate was found to be strain-dependent. Similar to LAB-1, several branched-chain and aromatic amino acids such as valine, leucine, isoleucine, tryptophan, tyrosine and phenylalanine appeared to be essential. Likewise, most of the strains lacked enzyme for the synthesis of 4-aminobutyric acid from glutamate.

### 3.6 Transport systems

The LAB**-**1 genome possesses genes for a wide range of transport systems. In total 151 transporters could be identified which belonged to at least six transport super families (Supplementary ∗∗S6). A large number (69) of the transporters were assigned to ATP-binding cassette (ABC) superfamily which includes many amino acid transporters such as glutamine, cysteine, and D-methionine transporters, putative tryptophan/tyrosine transporters, putative S-methylcysteine transporters etc. These amino acid transporters were basically substrate-binding proteins, ATP-binding proteins, or permeases. Some amino acid transporters from the electrochemical potential-driven transport family were also identified such as S-methylmethionine transporter, arginine:ornithine antiporter/lysine permease, and proton glutamate symport protein. Interestingly, the amino acid uptake systems were found to be larger in number than the sugar uptake systems.

Another important group of transporters, the major facilitator superfamily (MFS), was also predominant in the genome. MFS and ABC transport superfamilies are the two largest and most functionally versatile transport systems ubiquitously found in all organisms [52]. In LAB**-**1, the MFS transport system was represented by several drug transporters, a few organic acid transporters, phosphate and organophosphate transporters, nitrate/nitrite transporters etc. The drug transporters include multidrug resistance (MDR) proteins from the Drug:H+ antiporter-1 (DHA-1) family and one lincomycin resistance protein from DHA-2 family. The MFS type of MDR transporters are among the most abundant drug efflux systems in prokaryotes [53].

As for sugar transport, several components of the phosphotransferase system (PTS) were identified. PTS is one of the major active sugar transport systems in prokaryotes. The LAB-1 genome encodes two copies of “general component” of the PTS system called PTS enzyme I (PTSI; EC:2.7.3.9). PTSI is not sugar specific and catalyzes phosphorylation of various sugars with their concomitant translocation across the membrane. The bacterium also possesses several sugar specific PTS components. These include the sucrose specific PTS EIIBCA/EIIBC component; β-glucosidase specific EIICBA; cellobiose specific EIIC; galactitol specific EIIA, EIIB, and EIIC; and mannose specific EIIAB, EIIC, and EIID components. An advantage of the PTS system is that it is energetically more efficient than other active transport systems because nutrient transport in the PTS system is coupled with substrate level phosphorylation that increases its energy efficiency [41]. Sugar transporters of other types than PTS were also detected such as the lactose/raffinose/galactose permease, L-fucose permease, maltose/moltooligosaccharide transporter, and the glucose uptake protein glcU. The preponderance of transporters for the diverse range of sugars highlights the bacterium's capacity to metabolize a wide variety of carbohydrates from different sources.

### 3.7 Anti-phage defense systems

Phage infection is known to cause major problems for bacterial cultures in food, pharmaceutical, chemical and pesticide factories [54]. In food industry, it is the main cause of slow or failed fermentation, reduced acid production and reduced product quality [55]. Bacteria have evolved two vital DNA-level anti-phage systems to resist phage infections: restriction-modification (R-M) and Clustered Regularly Interspaced Short Palindromic Repeats (CRISPR)-CRISPR-associated (Cas) systems [56]. CRISPR-Cas, in addition to providing adaptive immunity against phages and foreign genetic elements, also confer additional biological effects beyond immunity [57]. In LAB-1, five genes of the R-M system were recognized which encoded the AlwI family type II restriction endonuclease, site-specific DNA-methyltransferase (adenine-specific), DNA (cytosine-5)-methyltransferase, Eco57I restriction-modification methylase domain-containing protein and Mrr type IV restriction system protein. Moreover, two CRISPR-Cas loci: 3507 bp having 28 direct repeats (DRs) and 57 spacers, and 472 bp having 32 DRs and 7 spacers, were also detected. Another important bacterial self-defense system is the presence of prophages that integrate into bacterial genome and confer resistance to phage superinfection [58]. Prophages also provide fitness benefits to bacteria via the transfer of beneficial genes [59]. Two prophage regions, one intact and another incomplete, were identified in LAB-1. The intact prophage region was 23 kb with 48.42% GC content. It had a total of 28 proteins including 25 phage proteins and 3 hypothetical proteins. Summarizing, LAB-1 appeared to have acquired multiple defense mechanisms to protect itself from plasmid and phage infections.

### 3.8 Stress response

**Figure 6:**
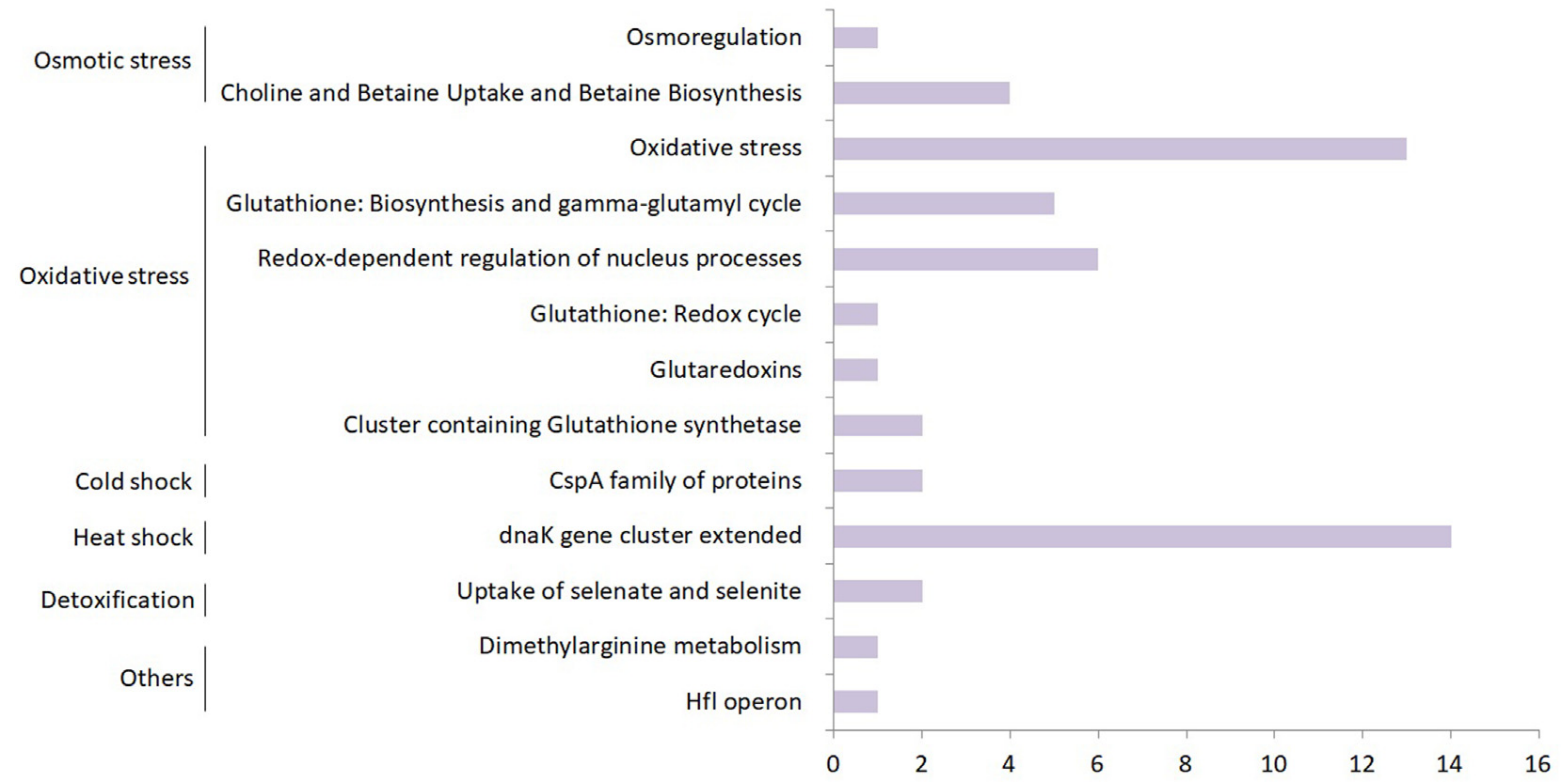
Subsystem feature counts of stress response in *L. fermentum* LAB-1.

LAB**-**1 genome carries a large number of genes contributing to osmotic, oxidative, cold, and heat stress resistance (Figure 6; Supplementary ∗∗S7). A diverse collection of chaperones and other proteins were present such as GroES, GroEL, GrpE, Hsp33, Hsp20, DnaK, DnaJ, HrcA, SmpB, YabA, LepA, RdgB etc. facilitating maintenance of protein integrity under the heat stress conditions. Two stress response proteins, CspA and CspB, which play major roles in bacterial responses to low temperatures, were found. Genes for the uptake of glycine betaine, glycerol and choline were present to support the organism's adaptation to osmotic stresses. The bacterium was also well equipped with genes for protection from oxidative stresses. It encodes potent antioxidants such as glutathione synthetase, thiol peroxidase, ferroxidase, disulphide oxidoreductase, few genes of the glutaredoxin system, and other antioxidant molecules to defend against oxidative stresses. Moreover, LAB**-**1 encodes Na^+^/H^+^ antiporters, ion transport channels, and proteins (Supplementary ∗∗S6) to protect itself from harsh environment like high salinity and high pH, the condition which is similar to that in the gut or dairy niches. Furthermore, the ability of exopolysaccharide production, as discussed later, can also support the organism to cope with high salinity, high pH and desiccation. In addition, genes of the arginine deiminase (ADI) pathway and amino acid decarboxylation reactions detected in the LAB-1 genome, might assist in its survival of pH, temperature and salinity stresses [43, 60, 61]. Previously, Vrancken et al. showed that ADI pathway helped the *L. fermentum* IMDO 130101 strain in adaptation to non-optimal growth conditions including temperature and added salt. Indeed, genomes of most lactic acid bacteria bear genes of the ADI and amino acid decarboxylation pathways which improve their resistance to acidic pH [43]. Additionally, genes encoding three other proteins involved in the biogenesis of cell envelope: 3-oxoacyl-synthase II, dTDP-glucose 4,6-dehydratase and dTDP-4-dehydrorhamnose 3,5-epimerase that were suggested to be key factors of intrinsic acid tolerance in *Lactobacillus plantarum* [62], were also present in LAB-1. Hence, LAB**-**1 is likely to survive in diverse conditions of the host-gut, or during industrial processing.

### 3.9 Vitamin biosynthesis

Due to the inability of human cells to synthesis 13 essential vitamins, an exogenous supply of these micronutrients is inevitable. Hence, consumption of foods enriched with vitamin**-**producing microbes can be of high benefits, especially in the deficiency conditions. In this context, the LAB**-**1 genome was screened for the genes of vitamin biosynthesis and those related to the production of several B-group vitamins and K vitamins were identified by RAST analysis (Supplementary ∗∗S8), for example, thiamine (vitamin B_1_), riboflavin (vitamin B_2_), pyridoxin (vitamin B_6_), biotin (vitamin B_7_), folate (vitamin B_9_), phylloquinone (vitamin K), and menaquinone (vitamin K_2_). Verce et al. also reported detection of all or most of the genes required for the biosynthesis of vitamin B_1_, coenzyme A, and nicotinic acid as well as thiamine salvage pathway in the core genome of several *Lactobacillus fermentum* strains [51]. *L. fermentum* 3872 was also reported to have genes required for the biosynthesis of B_1_, B_2_, B_5_, B_7_ and B_9_ [60]. The B group vitamins as well as vitamin K play essential functions in vital cellular processes such as DNA replication, repair, and methylation, amino acid and nucleotide synthesis etc. [63]. Consequently, their deficiencies, either due to inadequate dietary intake or inborn genetic disorders, may lead to major abnormalities. Although most essential vitamins can be obtained from foods, their deficiency is still found in many countries. The chemically synthesized pseudo-vitamins that are used to address the vitamin deficiencies do not have the same physiological effects as the natural vitamins produced by certain microorganisms [64]. Hence, instead of incorporating the chemical vitamins, foods can be naturally fortified with vitamins produced by the lactic acid bacteria to support the additional requirement. The food industries are expected to come forward to use the vitamin producing LAB in the production of vitamin bio-enriched foods which could be particularly beneficial to the vitamin-deficient populations.

### 3.10 Exopolysaccharide production

Genes for exopolysaccharide (EPS) biosynthesis were present in a cluster encoding the exopolysaccharide biosynthesis transcriptional activator (EpsA), tyrosine-protein kinase transmembrane modulator (EpsB), tyrosine-protein kinase (EpsC), protein-tyrosine phosphatase (EpsD), undecaprenyl-phosphate galactosephosphotransferase (EpsE), and glycosyltransferases. EPS production by *Lactobacillus* including a few *L. fermentum* strains has been described and considered to be a unique feature of the this genus [65]. Both in vitro study and genome characterization have previously indicated the production of EPS in *L. fermentum* including the strains KGC1601, 23271, ING8 etc [39, 40, 66]. Microbial EPS possess a wide spectrum of useful properties which have applications in food, pharmaceutical, nutraceutical, textile, chemical, cosmetics, agriculture, bioremediation and many other industries [67, 68]. In food industry, for example, EPS can act as texturizers and stabilizers, and improve the rheological and sensory properties of foods such as increased viscosity, thickness and emulsification, and reduced syneresis. In medicine, EPS can offer many health promoting effects such as antimicrobial, anti-viral, anti-inflammatory, antioxidant, immunomodulatory, anti-tumor, anti-ulcer, anti-diabetic, and cholesterol lowering effects [69]. EPS can also enhance probiotic competence of LAB. They protect cell walls from toxic compounds, protect cell integrity in harsh conditions, and provide resistance against antibiotics, bacteriophages, or phagocytosis [70]. Besides, EPS support microbial passage along gastrointestinal tract and their subsequent colonization in the gut. Enhancement of these probiotic competences further supports the expression of LAB's probiotic properties. EPS biosynthesis is, therefore, considered to be an important ability highly desired from the probiotic microbes. Indeed, most of the commercialized probiotics used today in biofunctional foods and therapeutics have this property.

### 3.11 Acetoin and butanediol production

Acetoin (3-hydroxybutan-2-one) and its reduced form, 2,3-butanediol, are flavor compounds that are in growing demands in the global market for their vast range of applications. Four genes related to their production were identified in the LAB**-**1 genome: (1) acetolactate synthase (catabolic; EC 2.2.1.6) which catalyzes the formation of acetolactate from pyruvate; (2) α-acetolactate decarboxylase (ALDC; EC 4.1.1.5) catalyzes decarboxylation of acetolactate into acetoin which is subsequently reduced into 2,3-butanediol in a reversible reaction by (3) 2,3-butanediol dehydrogenase (BDH; EC 1.1.1.4). Moreover, a fourth enzyme, acetoin (diacetyl) reductase (EC 1.1.1.304), which is involved in the irreversible reduction of diacetyl to acetoin could be detected. Both acetoin and butanediol achieved increasing importance in the production of chemicals, foods, fibers, cosmetics, plastics, resins and other industrial products [71]. Currently, their commercial production is mostly achieved by chemical process which is relatively inexpensive, but production based on biological systems has the advantage of being safer, ecofriendly and sustainable. Hence, the biobased butanediol market has been rapidly increasing and is estimated to grow further at a rate of 7.77 % per year over the period of 2021–2028 [72]. Besides, the recent interests in the new concept of biorefinery and the new wave of white biotechnology has opened up new avenues for developing an ecofriendly way to cater benefits from the microbe-mediated butanediol production using renewable biomasses [73].

### 3.12 Antimicrobial peptides

**Figure 7:**
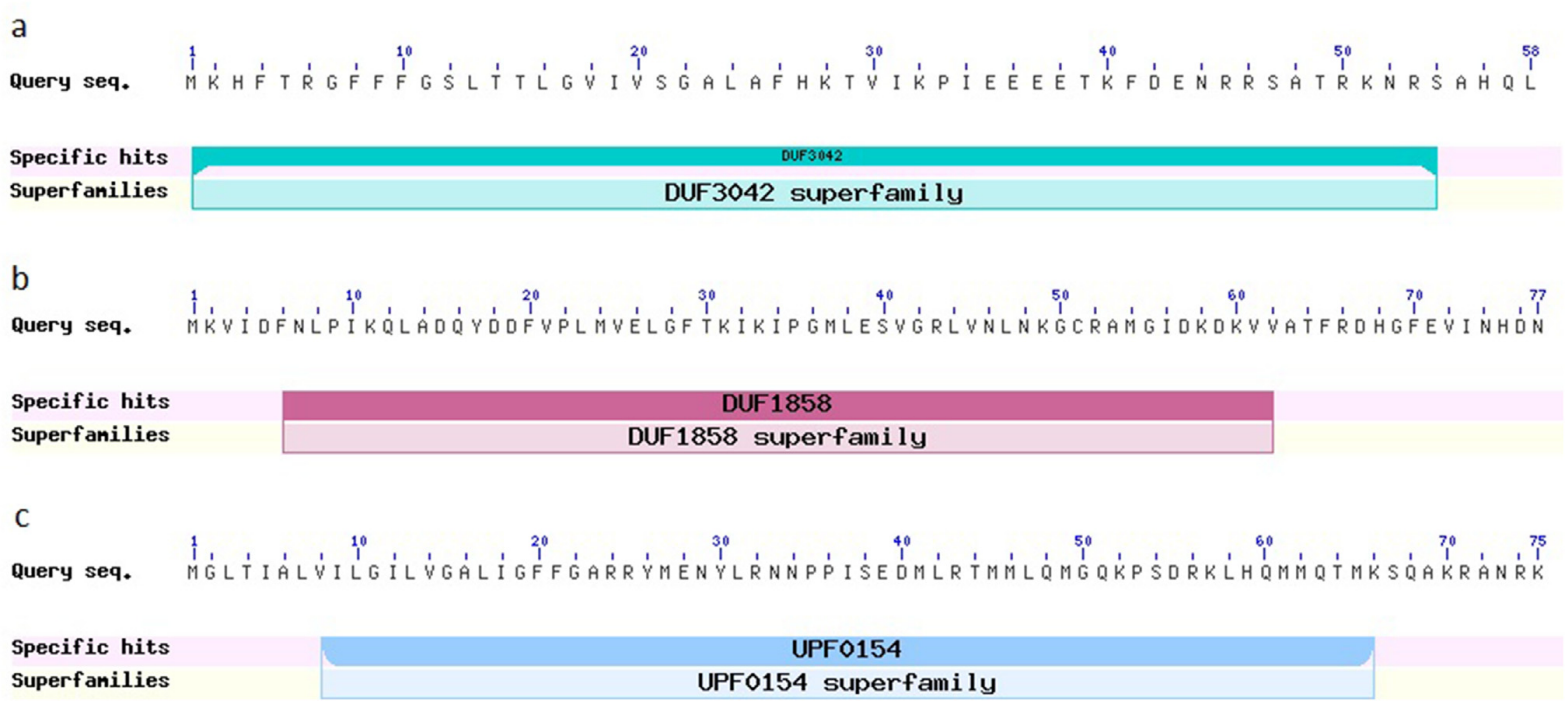
Conserved domains present in the three putative antimicrobial peptides identified in *L. fermentum* LAB-1. (a) WP_024271716.1; (b) WP_003683456.1; and (c) WP_003685231.1.

Functional annotations did not assign any of the LAB-1 genes to antimicrobial activity. Its antagonism of pathogenic bacteria [20], therefore, could be attributed mostly to its pH lowering effect by the secretion of organic acids. In fact, there appears to be only a few experimentally validated antimicrobial peptides reported in *L. fermentum*. Most recently, a gene originally annotated as a hypothetical protein in *L. fermentum* 23271 strain showed 48% sequence similarity to enterolysin A, a bacteriocin produced by *Enterococcus faecalis* which inhibited species of *Enterococcus, Lactobacillus, Lactococcus* and *Pediococcus* [38]. The functional genomics of the *L. fermentum* strain SK152 led to the identification of another putative antimicrobial gene encoding a putative endolysin [74]. Endolysins, commonly found in bacteriophages, exhibit a broad-spectrum antimicrobial activity. In a recent study, several putative bacteriocin-encoding genes were also identified in the *L. fermentum* LBM97 strain that were heterologously expressed in *Escherichia coli* [75]. Three of the expressed peptides showed antimicrobial activity in vitro against *E. coli* and/or *Staphylococcus aureus*. To find if identical antimicrobial peptides exist in LAB-1, sequences of the bacteriocins were used as query in BLAST searches conducted against the LAB-1 protein dataset (Table 1). Three peptides were found that showed over 98% sequence identity to the experimentally confirmed bacteriocins. This suggests a high probability of the identified peptides of LAB-1 to also exhibit similar antimicrobial effects. Conserved domain scan of the three peptides returned specific hits against complete DUF3042 (pfam11240), DUF1858 (pfam08984), or UPF0154 (pfam03672) domains (Figure 7). According to Pfam [76], functional roles of these domains are yet to be characterized. It is possible that these domains might play a role in the antimicrobial activity of the peptides.

**Table 1:** Sequence identity of LAB-1 peptides with bacteriocins.

LAB-1	Bacteriocin	Query cover (%)	E-value	Identity (%)
WP_024271716.1	BAG27613.1	100	2.00E−42	98.28
WP_003683456.1	ARB00861.1	100	6.00E−58	100
WP_003685231.1	EEI22241.1	100	1.00E−55	98. 67%

### 3.13 Safety concerns

Antibiotic resistance in probiotic or starter culture is extremely unsafe due to the risk of horizontal transfer of resistant genes to other microbes. But in many lactic acid bacteria the antibiotic resistance is intrinsic and, therefore, not transferable [77]. The LAB-1 strain was subjected to genomic evaluation of strain safety which did not identify any gene encoding acquired antimicrobial resistance suggesting that the bacterium can be considered safe concerning the spread of drug resistance. Moreover, genomic assessment of pathogenicity did not find any toxin, pathogenicity or virulence genes. The probability of LAB-1 being a human pathogen was estimated to be 0.087 with no matched pathogenic families, indicating a very low probability for LAB-1 to confer pathogenicity in human. Indeed, *L. fermentum* species can be generally considered safe in these respects and, like LAB-1, the underlying genetic basis has been demonstrated by the absence of transferable antibiotic resistant genes or pathogenicity and virulence genes in other strains as well [39, 40, 66].

## 4 Conclusions

Selection of appropriate microbe is the most important aspect in developing probiotic-enriched products. This necessitates systematic characterization of the candidate-microbe's probiotic properties. Genome analysis facilitates full elucidation of these properties at the genetic level. Functional characterization of LAB-1 genome in the present study revealed valuable genes of probiotic and industrial value. It has been found that LAB-1 has genes encoding nutraceutical products for health and biotechnological benefits, fermentation products for enhanced food quality, stress inducible proteins for survival in the gut and industrial conditions, defense systems for protection against phage infections; but the strain is devoid of genes encoding acquired drug resistance, pathogenesis or virulence. Although characterization of the above probiotic and physiological properties is based on the isolate's gene-functional analysis, laboratory research validating these beneficial traits is also underway. Furthermore, *L*. *fermentum* species has an additional advantage of holding the “Generally Recognized as Safe” (GRAS) status given by FAO. With its strong antimicrobial activity already proven against several pathogens, the isolate appears to be one of the most ideal probiotic-candidates to deliver multiple beneficial effects via functional foods and medicines.

## Declarations

### Author contribution statement

Tanim Jabid Hossain: Conceived and designed the experiments; Performed the experiments; Analyzed and interpreted the data; Contributed reagents, materials, analysis tools or data; Wrote the paper.

### Funding statement

This research did not receive any specific grant from funding agencies in the public, commercial, or not-for-profit sectors.

### Data availability statement

Data associated with this study has been deposited at GenBank under the accession number JAJTII000000000.1.

### Declaration of interest's statement

The authors declare no conflict of interest.

### Additional information

No additional information is available for this paper.

## Appendix A Supplementary data

Subsystems and Features.xlsx Subsystems and Features.xlsx

KEGG Mapper Reconstruction.xlsx KEGG Mapper Reconstruction.xlsx

Central Carbohydrate Metabolism.xlsx Central Carbohydrate Metabolism.xlsx

Carbohydrate-Active enZymes.xlsx Carbohydrate-Active enZymes.xlsx

Peptidases.xlsx Peptidases.xlsx

Transporters.xlsx Transporters.xlsx

Stress Response.xlsx Stress Response.xlsx

Cofactors, Vitamins.xlsx Cofactors, Vitamins.xlsx
